# 3-Fluoro-12*H*-benzimidazo[2,1-*b*][1,3]benzothia­zin-12-one

**DOI:** 10.1107/S1600536811054948

**Published:** 2012-01-14

**Authors:** Zhiming Wang, Bin Yu, Xiuqin Zhang, Yuan Cui, Xiaoqiang Sun

**Affiliations:** a School of Petrochemical Engineering, Changzhou University, Changzhou, Jiangsu 213164, People’s Republic of China; b High Technology Research Institute of Nanjing University, Changzhou 213162, People’s Republic of China

## Abstract

In the title compound, C_14_H_7_FN_2_OS, prepared by the reaction of 2-bromo-4-fluoro­benzoyl choride with 2-mercaptobenzimidazole, the four-membered fused-ring system is essentially planar [maximum deviation from the mean plane = 0.035 (2) Å]. The crystal packing is stabilized by weak inter­molecular π–π [minimum ring centroid–centroid separation = 3.509 (7) Å], weak C—F⋯π [F⋯centroid = 3.4464 (17) Å, C—F⋯centroid = 97.72 (11)°] and C—O⋯π [O⋯centroid = 3.5230 (16) and 3.7296 (17) Å, C—O⋯centroid = 86.40 (10) and 86.25 (10)°] inter­actions and weak inter­molecular C—H⋯N hydrogen bonds.

## Related literature

For general background to spiranes, see: Dawood & Abdel-Wahab (2010 ▶); Dolbier *et al.* (1994 ▶); Mavrova *et al.* (2010 ▶); Sekar *et al.* (2011 ▶).
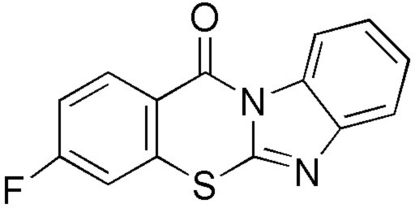


## Experimental

### 

#### Crystal data

C_14_H_7_FN_2_OS
*M* *_r_* = 270.28
Monoclinic, 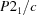

*a* = 9.5027 (12) Å
*b* = 7.0759 (9) Å
*c* = 16.931 (2) Å
β = 94.375 (3)°
*V* = 1135.1 (2) Å^3^
*Z* = 4
Mo *K*α radiation
μ = 0.29 mm^−1^
*T* = 293 K
0.20 × 0.18 × 0.15 mm

#### Data collection

Bruker SMART CCD area-detector diffractometer
Absorption correction: multi-scan (*SADABS*; Bruker, 2000 ▶) *T*_min_ = 0.944, *T*_max_ = 0.958
5987 measured reflections
1989 independent reflections
1772 reflections with *I* > 2σ(*I*)
*R*_int_ = 0.031

#### Refinement

*R*[*F* ^2^ > 2σ(*F* ^2^)] = 0.036
*wR*(*F* ^2^) = 0.106
*S* = 1.00
1989 reflections
172 parameters
H-atom parameters constrained
Δρ_max_ = 0.31 e Å^−3^
Δρ_min_ = −0.33 e Å^−3^

### 

Data collection: *SMART* (Bruker, 2000 ▶); cell refinement: *SAINT* (Bruker, 2000 ▶); data reduction: *SAINT*; program(s) used to solve structure: *SHELXTL* (Sheldrick, 2008 ▶); program(s) used to refine structure: *SHELXTL*; molecular graphics: *SHELXTL*; software used to prepare material for publication: *SHELXTL*.

## Supplementary Material

Crystal structure: contains datablock(s) I, global. DOI: 10.1107/S1600536811054948/ds2160sup1.cif

Structure factors: contains datablock(s) I. DOI: 10.1107/S1600536811054948/ds2160Isup2.hkl

Supplementary material file. DOI: 10.1107/S1600536811054948/ds2160Isup3.cml

Additional supplementary materials:  crystallographic information; 3D view; checkCIF report

## Figures and Tables

**Table 1:** Hydrogen-bond geometry (Å, °)

*D*—H⋯*A*	*D*—H	H⋯*A*	*D*⋯*A*	*D*—H⋯*A*
C14—H14⋯N1^i^	0.93	2.58	3.359 (2)	141

Symmetry code: (i) 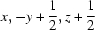.

**Fig. 1.**
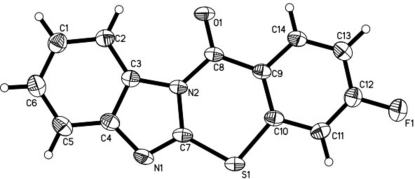
Ellipsoid plot.

## supplementary crystallographic information

### Comment

The chemistry of 2-mercaptoimidazole or 2-mercaptobenzimidazole has attracted much attention of many synthetic chemists owing to the occurrence of these ring systems in various biologically important compounds (Dawood & Abdel-Wahab, 2010; Mavrova *et al.*, 2010). In the past decades, most of these investigations were carried out with imidazole derivatives (Dolbier *et al.*, 1994; Sekar *et al.*, 2011). We herein present the structure of the title compound C_14_H_7_FN_2_OS, prepared from the reaction of 2-bromo-4-fluorobenzoyl choride with 2-mercaptobenzimidazole.

In the crystal structure, the title compound adopts an essentially planar conformation (Fig. 1), with the maximum atom deviation from the least-squares plane to the four-membered fused-ring system = 0.035 (2) Å. The dihedral angles between the benzimidazole ring (N1–C7) and thiazine ring (S1–C10) = 0.74 (8)°, the benzene ring (C9–C14) and thiazine ring (S1–C10) = 1.00 (4)° and the benzimidazole ring (N1–C7) and benzene ring (C9–C14) = 0.03 (8)°.

The crystal packing is stabilized by weak interactions: (1) intermolecular π–π interactions: (*a*) imidazole ring N1–C7 (ring 1) and benzene ring C1–C6 (ring 2) of the benzimazole moiety [ring centroid separation = 3.673 (8) Å, symmetry code (i)*-x*+1,*-y*+2, *-z*]; (*b*) between thiazine ring S1–C10 (ring 3) and S1–C10^i^ = 3.856 (5) Å; (*c*) between ring 3···and ring 2^i^ = 3.509 (7) Å; (2) C—O···π interactions [C(13)—O(1)···*Cg2*, C(13)—O(1)···*Cg3*] and C—F···π interactions [C(2)—F(1)···*Cg4*]; (3) intermolecular C—H···N hydrogen bonds [C(6)—H(6)···N(2)].

### Experimental

An oven-dried Schlenk tube was charged with a magnetic stirring bar, CuI (0.05 mmol), 1,10-phenanthroline (0.10 mmol), Cs_2_CO_3_ (0.50 mmol), and 2-mercaptobenzimidazole. The Schlenk tube was capped, and then evacuated and backfilled with N_2_ (3 times), then under a positive pressure of N_2_, a solution of 2-bromo-4-fluorobenzoyl choride (0.75 mmol) in touene (2 ml, freshly distilled from sodium) was added dropwise *via* syringe, and the mixture was pre-stirred for 1 h at room temperature. The reaction mixture was then stirred at 100 °C. After the reaction was completed, the mixture was cooled to room temperature, passed through Celite and rinsed with 30 ml of CH_2_Cl_2_. The combined filtrate was concentrated and purified by flash chromatography to give a white solid (93% yield). Single crystals of the title compound suitable for X-ray diffraction were obtained by evaporation of a petroleum ether–chloroform solution.

### Refinement

All the H atoms were placed in geometrically idealized positions and constrained to ride on their parent atoms, with C—H = 0.93 Å, with *U_iso_*(H) = 1.2 *U_eq_*(C)

### Crystal data

**Table:**

C_14_H_7_FN_2_OS	*F*(000) = 552
*M**_r_* = 270.28	*D*_x_ = 1.582 Mg m^−^^3^
Monoclinic, *P*2_1_/*c*	Mo *K*α radiation, λ = 0.71073 Å
Hall symbol: -P 2ybc	Cell parameters from 3848 reflections
*a* = 9.5027 (12) Å	θ = 2.4–29.8°
*b* = 7.0759 (9) Å	µ = 0.29 mm^−^^1^
*c* = 16.931 (2) Å	*T* = 293 K
β = 94.375 (3)°	Block, colourless
*V* = 1135.1 (2) Å^3^	0.20 × 0.18 × 0.15 mm
*Z* = 4	

### Data collection

**Table:**

Bruker SMART CCD area-detector diffractometer	1989 independent reflections
Radiation source: fine-focus sealed tube	1772 reflections with *I* > 2σ(*I*)
graphite	*R*_int_ = 0.031
phi and ω scans	θ_max_ = 25.0°, θ_min_ = 2.4°
Absorption correction: multi-scan (*SADABS*; Bruker, 2000)	*h* = −9→11
*T*_min_ = 0.944, *T*_max_ = 0.958	*k* = −8→8
5987 measured reflections	*l* = −20→18

### Refinement

**Table:**

Refinement on *F*^2^	Primary atom site location: structure-invariant direct methods
Least-squares matrix: full	Secondary atom site location: difference Fourier map
*R*[*F*^2^ > 2σ(*F*^2^)] = 0.036	Hydrogen site location: inferred from neighbouring sites
*wR*(*F*^2^) = 0.106	H-atom parameters constrained
*S* = 1.00	*w* = 1/[σ^2^(*F*_o_^2^) + (0.0611*P*)^2^ + 0.4006*P*] where *P* = (*F*_o_^2^ + 2*F*_c_^2^)/3
1989 reflections	(Δ/σ)_max_ < 0.001
172 parameters	Δρ_max_ = 0.31 e Å^−^^3^
0 restraints	Δρ_min_ = −0.33 e Å^−^^3^

### Special details

**Table:**

Geometry. All e.s.d.'s (except the e.s.d. in the dihedral angle between two l.s. planes) are estimated using the full covariance matrix. The cell e.s.d.'s are taken into account individually in the estimation of e.s.d.'s in distances, angles and torsion angles; correlations between e.s.d.'s in cell parameters are only used when they are defined by crystal symmetry. An approximate (isotropic) treatment of cell e.s.d.'s is used for estimating e.s.d.'s involving l.s. planes.
Refinement. Refinement of *F*^2^ against ALL reflections. The weighted *R*-factor *wR* and goodness of fit *S* are based on *F*^2^, conventional *R*-factors *R* are based on *F*, with *F* set to zero for negative *F*^2^. The threshold expression of *F*^2^ > σ(*F*^2^) is used only for calculating *R*-factors(gt) *etc*. and is not relevant to the choice of reflections for refinement. *R*-factors based on *F*^2^ are statistically about twice as large as those based on *F*, and *R*- factors based on ALL data will be even larger.

### Fractional atomic coordinates and isotropic or equivalent isotropic displacement parameters (Å^2^)

**Table:**

	*x*	*y*	*z*	*U*_iso_*/*U*_eq_	
S1	−0.14487 (5)	0.16939 (8)	0.36622 (3)	0.0538 (2)	
F1	−0.43167 (13)	0.1082 (2)	0.59933 (8)	0.0691 (4)	
O1	0.19345 (15)	0.3338 (2)	0.54945 (8)	0.0550 (4)	
N1	0.08266 (18)	0.2319 (2)	0.28889 (9)	0.0512 (4)	
N2	0.12490 (15)	0.2778 (2)	0.42060 (8)	0.0386 (3)	
C1	0.4856 (2)	0.4053 (3)	0.36667 (13)	0.0549 (5)	
H1	0.5742	0.4478	0.3860	0.066*	
C2	0.3838 (2)	0.3749 (3)	0.41907 (12)	0.0478 (4)	
H2	0.4024	0.3929	0.4733	0.057*	
C3	0.2520 (2)	0.3161 (2)	0.38695 (11)	0.0409 (4)	
C4	0.2239 (2)	0.2870 (3)	0.30570 (11)	0.0458 (4)	
C5	0.3284 (2)	0.3134 (3)	0.25403 (12)	0.0570 (5)	
H5	0.3112	0.2914	0.2000	0.068*	
C6	0.4596 (2)	0.3741 (3)	0.28586 (13)	0.0600 (5)	
H6	0.5314	0.3942	0.2525	0.072*	
C7	0.0288 (2)	0.2291 (2)	0.35679 (10)	0.0429 (4)	
C8	0.0987 (2)	0.2875 (2)	0.50112 (10)	0.0404 (4)	
C9	−0.04393 (18)	0.2395 (2)	0.52280 (10)	0.0387 (4)	
C10	−0.1557 (2)	0.1855 (2)	0.46872 (11)	0.0420 (4)	
C11	−0.2867 (2)	0.1387 (3)	0.49526 (12)	0.0474 (5)	
H11	−0.3609	0.0999	0.4599	0.057*	
C12	−0.3033 (2)	0.1512 (3)	0.57463 (12)	0.0496 (5)	
C13	−0.1972 (2)	0.2037 (3)	0.62965 (12)	0.0517 (5)	
H13	−0.2121	0.2090	0.6833	0.062*	
C14	−0.0687 (2)	0.2480 (3)	0.60323 (11)	0.0446 (4)	
H14	0.0043	0.2848	0.6397	0.054*	

### Atomic displacement parameters (Å^2^)

**Table:**

	*U*^11^	*U*^22^	*U*^33^	*U*^12^	*U*^13^	*U*^23^
S1	0.0482 (3)	0.0744 (4)	0.0365 (3)	−0.0045 (2)	−0.0113 (2)	−0.0035 (2)
F1	0.0542 (7)	0.0824 (9)	0.0723 (8)	−0.0065 (6)	0.0150 (6)	−0.0017 (7)
O1	0.0544 (8)	0.0695 (9)	0.0387 (7)	−0.0113 (7)	−0.0121 (6)	−0.0058 (6)
N1	0.0585 (10)	0.0611 (10)	0.0325 (8)	0.0036 (8)	−0.0060 (7)	0.0026 (7)
N2	0.0429 (8)	0.0397 (8)	0.0319 (7)	0.0020 (6)	−0.0068 (6)	−0.0009 (6)
C1	0.0490 (11)	0.0546 (11)	0.0610 (12)	0.0014 (9)	0.0031 (9)	0.0037 (9)
C2	0.0474 (10)	0.0477 (10)	0.0475 (10)	0.0006 (8)	−0.0014 (8)	−0.0001 (8)
C3	0.0469 (10)	0.0344 (8)	0.0407 (9)	0.0051 (7)	−0.0021 (8)	0.0031 (7)
C4	0.0537 (11)	0.0430 (9)	0.0399 (10)	0.0074 (8)	−0.0024 (8)	0.0059 (8)
C5	0.0690 (14)	0.0596 (12)	0.0425 (11)	0.0118 (10)	0.0046 (10)	0.0067 (9)
C6	0.0596 (13)	0.0646 (13)	0.0573 (13)	0.0074 (10)	0.0135 (10)	0.0108 (10)
C7	0.0491 (10)	0.0412 (9)	0.0362 (9)	0.0041 (8)	−0.0105 (7)	0.0007 (7)
C8	0.0480 (10)	0.0377 (9)	0.0341 (9)	0.0022 (7)	−0.0070 (7)	−0.0009 (7)
C9	0.0448 (10)	0.0336 (8)	0.0365 (9)	0.0050 (7)	−0.0052 (7)	0.0002 (7)
C10	0.0488 (10)	0.0368 (9)	0.0389 (9)	0.0059 (7)	−0.0066 (8)	0.0012 (7)
C11	0.0431 (10)	0.0449 (10)	0.0525 (11)	0.0021 (8)	−0.0075 (8)	0.0003 (8)
C12	0.0473 (11)	0.0447 (10)	0.0572 (12)	0.0039 (8)	0.0069 (9)	0.0030 (8)
C13	0.0619 (12)	0.0507 (11)	0.0425 (10)	0.0065 (9)	0.0053 (9)	−0.0003 (8)
C14	0.0507 (10)	0.0443 (9)	0.0378 (9)	0.0050 (8)	−0.0046 (8)	−0.0012 (7)

### Geometric parameters (Å, °)

**Table:**

S1—C7	1.723 (2)	C4—C5	1.385 (3)
S1—C10	1.7501 (19)	C5—C6	1.388 (3)
F1—C12	1.354 (2)	C5—H5	0.9300
O1—C8	1.214 (2)	C6—H6	0.9300
N1—C7	1.294 (2)	C8—C9	1.471 (3)
N1—C4	1.406 (3)	C9—C10	1.402 (2)
N2—C3	1.401 (2)	C9—C14	1.401 (2)
N2—C7	1.403 (2)	C10—C11	1.395 (3)
N2—C8	1.406 (2)	C11—C12	1.368 (3)
C1—C2	1.378 (3)	C11—H11	0.9300
C1—C6	1.389 (3)	C12—C13	1.371 (3)
C1—H1	0.9300	C13—C14	1.369 (3)
C2—C3	1.390 (3)	C13—H13	0.9300
C2—H2	0.9300	C14—H14	0.9300
C3—C4	1.397 (3)		
			
C7—S1—C10	101.85 (9)	N1—C7—S1	122.20 (14)
C7—N1—C4	105.11 (16)	N2—C7—S1	124.09 (14)
C3—N2—C7	105.38 (14)	O1—C8—N2	119.32 (17)
C3—N2—C8	127.34 (15)	O1—C8—C9	122.96 (16)
C7—N2—C8	127.28 (15)	N2—C8—C9	117.72 (15)
C2—C1—C6	121.9 (2)	C10—C9—C14	118.16 (17)
C2—C1—H1	119.1	C10—C9—C8	124.53 (16)
C6—C1—H1	119.1	C14—C9—C8	117.31 (16)
C1—C2—C3	116.76 (19)	C11—C10—C9	120.31 (17)
C1—C2—H2	121.6	C11—C10—S1	115.18 (14)
C3—C2—H2	121.6	C9—C10—S1	124.51 (15)
C2—C3—C4	121.90 (18)	C12—C11—C10	118.25 (18)
C2—C3—N2	132.68 (17)	C12—C11—H11	120.9
C4—C3—N2	105.41 (16)	C10—C11—H11	120.9
C5—C4—C3	120.66 (19)	F1—C12—C13	118.95 (18)
C5—C4—N1	128.96 (19)	F1—C12—C11	117.57 (18)
C3—C4—N1	110.38 (17)	C13—C12—C11	123.47 (19)
C4—C5—C6	117.5 (2)	C12—C13—C14	117.93 (19)
C4—C5—H5	121.2	C12—C13—H13	121.0
C6—C5—H5	121.2	C14—C13—H13	121.0
C5—C6—C1	121.2 (2)	C13—C14—C9	121.85 (18)
C5—C6—H6	119.4	C13—C14—H14	119.1
C1—C6—H6	119.4	C9—C14—H14	119.1
N1—C7—N2	113.71 (17)		
			
C6—C1—C2—C3	−1.7 (3)	C10—S1—C7—N2	−0.75 (17)
C1—C2—C3—C4	0.7 (3)	C3—N2—C8—O1	0.0 (3)
C1—C2—C3—N2	−177.85 (18)	C7—N2—C8—O1	−179.37 (17)
C7—N2—C3—C2	178.39 (19)	C3—N2—C8—C9	−179.66 (15)
C8—N2—C3—C2	−1.1 (3)	C7—N2—C8—C9	0.9 (2)
C7—N2—C3—C4	−0.31 (18)	O1—C8—C9—C10	180.00 (17)
C8—N2—C3—C4	−179.83 (15)	N2—C8—C9—C10	−0.3 (2)
C2—C3—C4—C5	1.0 (3)	O1—C8—C9—C14	−0.2 (3)
N2—C3—C4—C5	179.83 (16)	N2—C8—C9—C14	179.46 (15)
C2—C3—C4—N1	−178.82 (16)	C14—C9—C10—C11	−1.1 (2)
N2—C3—C4—N1	0.06 (19)	C8—C9—C10—C11	178.63 (16)
C7—N1—C4—C5	−179.51 (19)	C14—C9—C10—S1	179.32 (13)
C7—N1—C4—C3	0.2 (2)	C8—C9—C10—S1	−0.9 (2)
C3—C4—C5—C6	−1.6 (3)	C7—S1—C10—C11	−178.27 (13)
N1—C4—C5—C6	178.15 (19)	C7—S1—C10—C9	1.29 (17)
C4—C5—C6—C1	0.6 (3)	C9—C10—C11—C12	1.5 (3)
C2—C1—C6—C5	1.0 (3)	S1—C10—C11—C12	−178.94 (14)
C4—N1—C7—N2	−0.5 (2)	C10—C11—C12—F1	178.98 (16)
C4—N1—C7—S1	179.82 (13)	C10—C11—C12—C13	−1.4 (3)
C3—N2—C7—N1	0.5 (2)	F1—C12—C13—C14	−179.48 (17)
C8—N2—C7—N1	−179.97 (16)	C11—C12—C13—C14	0.9 (3)
C3—N2—C7—S1	−179.78 (13)	C12—C13—C14—C9	−0.5 (3)
C8—N2—C7—S1	−0.3 (2)	C10—C9—C14—C13	0.6 (3)
C10—S1—C7—N1	178.93 (15)	C8—C9—C14—C13	−179.15 (16)

### Hydrogen-bond geometry (Å, °)

**Table:**

*D*—H···*A*	*D*—H	H···*A*	*D*···*A*	*D*—H···*A*
C14—H14···N1^i^	0.93	2.58	3.359 (2)	141

Symmetry codes: (i) *x*, −*y*+1/2, *z*+1/2.
